# Evaluation of Electronic Mental Health Implementation in Northern Territory Services Using the Integrated “Promoting Action on Research Implementation in Health Services” Framework: Qualitative Study

**DOI:** 10.2196/14835

**Published:** 2020-05-26

**Authors:** Buaphrao Raphiphatthana, Michelle Sweet, Stefanie Puszka, Megan Whitty, Kylie Dingwall, Tricia Nagel

**Affiliations:** 1 Menzies School of Health Research Casuarina Australia; 2 Australian National University Canberra Australia

**Keywords:** eHealth, implementation science, health care delivery

## Abstract

**Background:**

Electronic mental health is a promising strategy to bridge the treatment gap in mental health care. Training workshops have been delivered to service providers working with Aboriginal and Torres Strait Islander people at a primary health care level to raise awareness and knowledge of electronic mental health approaches.

**Objective:**

This study aimed to understand service providers’ perspectives and experiences of electronic mental health adoption. More specifically, it aimed to use the integrated Promoting Action on Research Implementation in Health Services (i-PARIHS) framework to further identify and understand how different factors facilitate or impede electronic mental health uptake within primary health care settings providing services to Aboriginal and Torres Strait Islander people.

**Methods:**

Qualitative interviews were conducted with 57 service providers working with Aboriginal and Torres Strait Islander people, who had undergone electronic mental health training workshops.

**Results:**

Several factors related to innovation (electronic mental health approach), recipients (service providers as an individual and as a team), and context (local, organizational, and external contexts) were found to influence electronic mental health uptake. Particularly, organizational readiness, in terms of information technology resources and infrastructure, policies, workforce and culture, and processes to mandate electronic mental health use, were found to be significant impediments to electronic mental health utilization. These findings led to the development of a three-phase implementation strategy that aims to enhance electronic mental health adoption by addressing organizational readiness before and post electronic mental health training.

**Conclusions:**

The i-PARIHS provides a useful determinant framework that deepens our understanding of how different factors impede or facilitate electronic mental health adoption in this setting. This insight was used to develop a practical and comprehensive implementation strategy to enhance the utilization of electronic mental health approaches within primary health care settings, involving three phases: pretraining consultations, training workshops, and post-training follow-up support.

## Introduction

### Electronic Mental Health Background

It is estimated that 450 million people worldwide are affected by mental illness, that is, at least one in four people are burdened by mental health conditions. This places mental illness as one of the leading causes of ill health and disability worldwide [1]. Despite the high prevalence of mental illness, a significant proportion of burdened individuals are not receiving treatment [2-4]. The treatment gap is a global challenge involving a complex interplay between multiple factors from policy to individual elements and thus requires an innovative and feasible solution.

With the omnipresence of the internet and technology in today’s society, many scholars recognize the potential of digital media as a promising strategy to bridge the treatment gap in mental health care. This surge of interest led to the emergence of the electronic mental health field in 1999 [5]. An electronic mental health approach has been defined as “the use of information and communication technology... to support and improve mental health conditions and mental health care” [6]. Typically, the approach involves the use of a variety of digital platforms such as telephone, mobile, computer, and Web-based apps.

One of the attractions of the electronic mental health approach is flexibility. Electronic mental health can be applied at different levels of health care, such as promotion, prevention, early intervention, active treatment, maintenance, and relapse intervention. Its mode of utilization can also vary. Some tools are designed to be self-directed and used without professional support, whereas others are practitioner-guided [7,8]. Other strengths of electronic mental health include improved accessibility, cost-efficiency, interactivity, and consumer engagement [8,9].

Evidence for the effectiveness of electronic mental health in addressing mental health concerns has begun to emerge in the last couple of decades. Several systematic reviews and meta-analyses have shown that the approach is effective in reducing symptoms for a range of mental illnesses, for example, depression, anxiety, eating disorders, and substance misuse [10-12].

Researchers and clinicians have, however, raised some concerns about the approach. In particular, issues around cybersecurity, confidentiality, data storage, and management have been highlighted [13]. Other concerns include the level of responsivity to crises, the lack of guidelines, and the limited number of evidence-based tools and resources [14]. Nonetheless, efforts are underway to address these concerns. Several professional organizations have issued ethical guidelines for Web-based counseling [15,16] as well as for risk management of crises [17]. In Australia, electronic portals (ePortals) have been developed to direct individuals to electronic mental health tools and resources that have been filtered by experts, for example, HeadtoHealth and Beacon [18]. The Australian government has also launched a project to develop a certification framework to ensure the quality of electronic mental health resources and their safe application [19].

### Electronic Mental Health in Australia

In line with global statistics, the disparity between demand and supply of mental health services in Australia is significant [20]. To reduce the burden and treatment gap of mental illness in Australia, the Australian government released the National eMental Health Strategy, which aims to raise awareness and utilization of electronic mental health approaches by all Australians [21]. The initiative encompasses three components: the head to health website (ePortal), Mindspot (electronic clinic]), and nationwide electronic mental health training and implementation support (electronic mental health in practice [eMHPrac]).

Menzies School of Health Research (Menzies) is a partner in the eMHPrac project. In collaboration with the University Centre for Rural Health (UCRH) and Queensland University of Technology (QUT), we focus on raising awareness and promoting electronic mental health resources along with providing training and support to service providers working with Aboriginal and Torres Strait Islander people (First Nations hereafter) at the primary health care level, for example, nurse, peer support worker, Indigenous health worker, psychologist, and alcohol and other drug workers. In our use of the term First Nations, we acknowledge the diversity of Australian Aboriginal and Torres Strait Islander languages and traditions. The training package aims to educate service providers on available evidence-based electronic mental health resources and tools. The Menzies Aboriginal and Islander Mental Health Initiative (AIMhi) Stay Strong app is one of the relatively few available apps responsive to First Nations cultures.

### Electronic Mental Health Within Australia’s First Nations Context

For First Nations, positive identities, extensive social networks, connectivity with languages, and traditions can act as protective factors for well-being; nevertheless, there is a much higher prevalence of mental health concerns in comparison to other Australian communities [22]. The multiple underlying factors include consequences of colonization, marginalization, intergenerational trauma, and enduring inequality and racism [23,24]. Despite the high need for support, access to appropriate services is poor, particularly for those who live in remote areas [25]. With the rising presence of digital media in First Nations communities, particularly for youth [26,27], an electronic mental health approach has the potential to bridge the treatment gap and overcome the barriers to services such as stigma and geography.

Research on the application of electronic mental health in Australia First Nations contexts is still in its infancy; however, the approach is perceived to be feasible, acceptable, and appropriate [28]. From the service providers' perspective, the approach is deemed useful for rapport building and engagement with First Nations clients. Its applicability across a range of clients, services, and settings is perceived to be broad and viable [28]. Enthusiasm for electronic mental health is also expressed by community members. Povey et al [29] conducted focus groups exploring the response of First Nations community members to their experience of two culturally adapted apps. Overall, the community members were optimistic about electronic mental health and its potential for improving well-being for First Nations. The three key factors identified as influencing the acceptability of electronic mental health approaches within indigenous settings were as follows: personal factors (eg, motivation and information technology [IT] literacy), environmental factors (eg, availability of resources), and app characteristics (eg, graphics and content).

### Implementation of Electronic Mental Health

It takes approximately 17 years for research to be translated into clinical practice [30]. The time lag is referred to as the translation gap, and it is a forefront issue in health research [31]. Minimizing this gap optimizes patient benefits and the use of scarce resources. It is therefore imperative to gain an understanding of the factors influencing translation to inform robust implementation strategies.

To date, little is known regarding effective strategies for implementing electronic mental health resources in primary health care services working with Australia’s First Nations. Findings from the very few studies in this field report common themes essential to electronic mental health uptake relating to three key domains: organizational factors, characteristics of the adopters (ie, practitioners and clients), and the innovation itself. For example, factors that impose challenges to adoption are the lack of organizational policy and procedural support for electronic mental health utilization, and IT infrastructure and resources. Poor IT literacy and knowledge of electronic mental health also impedes the uptake of the electronic mental health approach. Utilization is also dependent on the characteristics of the innovation, that is, user-friendliness and cultural responsiveness [32,33].

### This Study

This study aimed to further the current knowledge of electronic mental health implementation in primary care services working with Australia's First Nations. A conceptual framework, the integrated Promoting Action on Research Implementation in Health Services (i-PARIHS), was used to deepen the understanding of factors influencing adoption. The framework applied here is a revised version of the Promoting Action on Research Implementation in Health Services (PARIHS) [34,35].

The *i*-PARIHS framework conceptualizes successful implementation as “achievement of agreed implementation/project goals, the uptake and embedding of the innovation in practice, individuals, teams and stakeholders are engaged, motivated, and ‘own’ the innovation, variation related to context is minimized across implementation settings” [34]. The framework posits that factors relating to innovation (*innovation*, I), the individuals involved in the implementation process (*recipients*, R), and the settings in which evidence-based practice is to be implemented (*context*, C) are key considerations for *facilitation* (Fac^n^), which drives successful implementation. In other words, *facilitation* is an active ingredient that assesses and draws knowledge from I, R, and C to inform strategies and processes to enable successful implementation.

The *i*-PARIHS is deemed useful as a determinant framework for our analysis, as it resonates with findings from our earlier work [33]. That is, factors related to the context, individuals, and innovation were used to inform implementation efforts. This study, therefore, used the *i*-PARIHS to retrospectively evaluate factors influencing electronic mental health adoption, with a focus on the core constructs, *context*, *innovation,* and *recipients*. By gaining a greater understanding of these three constructs in relation to electronic mental health implementation within primary health care services working with First Nations, we will be able to develop a tailored facilitation strategy for future electronic mental health implementation programs within this context.

For the purpose of this study, *innovation* is defined as the integration of external evidence (knowledge of research, clinical, and patients’ experience relating to electronic mental health approaches) with local priorities and practices to impact electronic mental health uptake*; recipients* refers to the actors involved in, affected by, and influencing implementation; *context* is the setting in which the proposed change is to be implemented and is considered across different levels from local organizational to the external health system [34].

The specific objectives of this study were threefold. First, to continue identifying important factors in the adoption of electronic mental health from the service providers’ perspective. Second, to further understand how they influence (ie, impede or facilitate) electronic mental health adoption by service providers working with First Nations, with reference to the *i*-PARIHS framework. Third, to evaluate the usefulness of the *i*-PARIHS framework in this specific context by determining which elements are helpful as theoretical guides and how they are operationalized in practice. From our findings, we aimed to further the current understanding of electronic mental health implementation and to provide practical guidance for future implementation strategies for services and researchers working with Australia’s First Nations.

## Methods

### The Role of Menzies School of Health Research

The eMHPrac project is a joint venture involving multiple organizations across Australia to support electronic mental health uptake using a number of dissemination and translation strategies, including Web-based campaigning, promotions at community activities and conferences, and provision of electronic mental health training. Menzies have extensive experience in developing and training service providers in culturally grounded mental health interventions through the AIMhi research program [36]. Therefore, our primary role in the project is to provide electronic mental health training workshops for service providers working with First Nations at the primary care level.

The training aims to raise awareness of validated electronic mental health tools and resources, specifically those that are responsive to First Nations cultures as well as to build service providers’ skills and confidence in applying them in their practice. The training workshops, adapted from the AIMhi training program *Yarning about Indigenous Mental Health* [37], are delivered by Indigenous and non-Indigenous trainers. An in-depth description of the training workshop is reported elsewhere [38].

### Participants and Settings

From October 2013 to December 2015, 261 participants received training, and 21 training workshops were held in Darwin, Alice Springs, and remote Northern Territory communities. The training workshops were advertised through professional networks, newsletters, and the Menzies website. Participants either self-selected or were selected by their organizations to attend the training. The participants were service providers working with First Nations at the primary care level, such as nurses, support workers, Indigenous health workers, psychologists, and alcohol and other drug workers.

Between June 2015 and November 2015, service providers who had completed the training program were approached for a follow-up interview via email and telephone. The aim was to interview as many trainees as possible; however, the final sample, 57, only represents a proportion of those trained. The main reason for nonparticipation was staff movement into different roles and organizations. The demographic information of the trainees who participated in the interview, that is, ethnicity, primary role, profession, and service type, is reported in Table 1. Professions reported in the *Other* category included support/administrative and managerial staff, nutritionists, and dieticians.

**Table 1:** Participants’ primary role, profession, and service type (N=57).

Participants		Values, n (%)	
**Primary role (n=53)**			
	Family well-being		2 (4)
	Social and emotional well-being		15 (28)
	Chronic disease		7 (13)
	Youth		12 (23)
	Alcohol and other drugs		4 (8)
	Primary care		1 (2)
	Other		12 (23)
**Profession (n=54)**			
	Aboriginal community worker		3 (6)
	Alcohol and other drug worker		3 (6)
	General nurse		5 (9)
	Mental health nurse		1 (2)
	Other nurse		2 (4)
	Psychologist		3 (6)
	Social worker		11 (20)
	Peer support worker		2 (4)
	Trainer/educator		1 (2)
	Manager/coordinator/chief executive officer		6 (11)
	Other		17 (32)
**Service type (n=53)**			
	Aboriginal community−controlled health service		8 (13)
	Nongovernment community		30 (57)
	Government community		13 (25)
	Other		2 (4)

### Research Design

A qualitative approach, embedded in a contextualist paradigm, was used to deepen our understanding of factors that influence, that is, impede or facilitate, uptake of electronic mental health approaches by primary care service working with First Nations. As explained by Braun and Clarke, contextualism is an epistemological position that sits between positivism and constructionism. This epistemology recognizes that knowledge is shaped by and acquires meaning in particular contexts, which may be physical, cultural, or temporal. Through a contextualist paradigm, we understand electronic mental health implementation as a process that is inalienable from local service provision contexts [39]. A semistructured interview was selected as the method to explore participants’ perceptions and experiences of using electronic mental health resources.

The research team was involved in the development of the Stay Strong app (which is included in the training workshops), delivery of the training workshops, follow-up support, and conducting the interviews. This limitation is emphasized in the Discussion section.

### Data Collection

The one-on-one, face-to-face, semistructured interviews conducted in Alice Springs were carried out by MS; those in Darwin were conducted by Katrina Kawaljenllo and SP. MS and SP are female researchers who conducted the training workshops and provided follow-up support. The interviews were held at the researchers’ or participants’ workplaces, except for three that were conducted over the phone as the participants worked in remote communities, that is, Groote Eylandt and Borroloola. The semistructured interview guide was developed by the research team based on the team’s knowledge of the implementation literature and experience researching and working with service providers in electronic mental health implementation. It was designed to explore participants’ attitudes and experiences with electronic mental health approaches, and the perceived real or potential implementation issues.

The interviews took approximately 45 min to an hour to complete. The interviews were recorded, and the audio was transcribed verbatim by a professional transcription service and then entered into the NVivo^2^ software program. The transcribed data were not returned to participants for comments. Participants’ confidentiality was assured by the assignment of ID codes. The extracts reported here are deidentified. All participants provided written consent to interviews and audio recordings, and ethics approval was granted by all relevant ethics committees (reference number HREC 12-1881 and CAHREC 12-100).

### Data Analysis

A thematic analysis was used to analyze the data. The analysis oscillated between deductive and inductive approaches and was carried out in two phases. The first phase involved familiarization with the data by multiple readings, and the preliminary coding of the data was conducted independently by 2 authors, MS and MW. At this stage, the codes were formed inductively, and common perspectives were grouped together with an assigned descriptive code name. The comparison revealed consistency across categories and broad agreement on codes and key themes. Data saturation was reached. Through collaboration, 3 members of the research team (MS, MW, and TN) reinterpreted participants’ experiences by using the original PARIHS framework to retrospectively evaluate key elements influencing electronic mental health implementation. The coded extracts were categorized into themes in reference to the elements and subelements of the framework through extensive discussion between the authors.

Post analysis, the *i*-PARIHS framework was discovered, and thus another phase of analysis took place to ensure that the most up-to-date conceptual framework was used. The key differences between the original PARIHS and the revised *i*-PARIHS framework is the addition of the *recipient* construct, expansion of the *context* element to encapsulate inner (local and organizational context) and outer context (the wider health system), and reconceptualization of *facilitation* to be an overarching active factor that assesses and responds to the other core constructs. The second phase of analysis involved recategorization of the preidentified data extracts from the first phase to the revised *i*-PARIHS framework elements and subelements (BR). Therefore, the final themes were established deductively based on the relevant principles of the *i*-PARIHS framework. The remapping of data extracts to the *i*-PARIHS elements and interpretation of the data were consulted with TN and MS to ensure rigor and validity. Owing to the time lag between the interviews and the final stages of analyses, the final findings were not reviewed by the participants. However, our findings will be disseminated through the newsletter, which will be sent to participants on the distribution list.

## Results

### Aims of the Analysis

The analysis aimed to identify factors and to form an understanding of how they impede or facilitate electronic mental health utilization. The *i*-PARIHS was used as a determinant framework, that is, as a descriptive purpose for identifying factors that influence implementation outcomes. Therefore, the core elements and subelements that are supported by data are presented according to the framework’s conceptualization. In addition, as previously discussed, the *facilitation* construct is not presented in the results, as it is considered to be the next phase of the implementation process.

The aim of the study was to explore the utilization of an electronic mental health approach in general; however, many of the responses were related to the use of the Stay Strong app. The app, developed as part of the AIMhi research program, is a strength-based brief intervention designed to incorporate both Indigenous and Western perspectives of mental health and well-being [36]. The target audience for the training workshops was service providers working with Indigenous Australians, and so a portion of the training was focused on electronic mental health apps that are culturally responsive to indigenous cultures. The Stay Strong app is one of the very few culturally adapted tools, and thus attracted a lot of interest from the trainees.

### Innovation

Definition: The integration of external evidence (knowledge of research, clinical, and patients’ experience relating to electronic mental health approaches) with local priorities and practice impact on electronic mental health uptake.

#### Underlying Knowledge Sources

The three key areas of knowledge relating to electronic mental health approaches identified as essential in the decision-making process to use a particular resource include the context in which the resource was developed, the population it was developed for, and whether it has been validated. Utilization was dependent on whether the user was confident that the information embedded in the resource aligned with the local settings and target populations. Some of the participants expressed a lack of knowledge and confusion regarding the context and the population for which the innovation was developed and validated. Therefore, they were uncertain about the suitability of the resources for their clients:

A lot of the time there are resources listed but then you are not really sure where they were developed, in what context, and then if they would be useful in the Central Australian context. Sometimes it could be more tailored towards Top End, or somewhere else completely.

P81, social worker, nongovernment community

#### Degree of Fit With Existing Practice and Values

The degree of change required to incorporate electronic mental health into current practice was perceived by a minority of the participants to influence the speed of adoption. For example, a participant said:

It just required us all to change practice a bit slightly too. So we dragged the ball a bit.

P129, peer support worker, government community

This, however, appears to vary across organizations as another participant reported that minimal change in practice was needed:

No change to clinical practice... we just incorporate into our framework of how we work with young people.

P32, social worker, nongovernment community

Uptake of electronic mental health approaches was dependent upon the perceived fit of the innovation to the organization’s priorities. Specific to the Stay Strong app, some participants reported that conducting brief interventions was not an immediate priority; hence, the app was not fully integrated into practice. Utilization was also dependent on the perceived fit with service providers’ roles and responsibilities. For example, a participant commented that alcohol and other drug workers perceived their primary responsibility to include the following but not providing brief interventions:

taking people to appointments for different social needs

P89, psychologist, Aboriginal community-controlled health service

Thus, the Stay Strong app was not incorporated into their practice. In addition, participants in a managerial role explained the nonutilization of eMental Health due to its irrelevance to the role (eg, acting manager).

Characteristics of the target population (age, culture, and IT literacy) were also important considerations. With regard to age, some participants expressed concerns over electronic mental health incompatibility with the older population:

the older people don’t really relate to the animation and to the touching of the screen. They don’t really feel comfortable.

P58, general nurse, Aboriginal community−controlled health service

The majority of the participants perceived electronic mental health to be applicable and suitable for young people, and so those who worked with youth were more receptive to the innovation.

I work with young people between twelve and eighteen and technology is their bible, you know the things they go to most. So I find that the use of electronic mental health is like a good resource that young people feel comfortable using.

P205, youth and family worker, nongovernment community

Electronic mental health was also perceived to be potentially beneficial and useful for First Nations communities:

A lot of the work we do is based on community needs and a very traditional way of doing things…but even they want more different ways of doing things as well and capturing information on film and photographs and applications like this... So I think there is a real need for communities to use applications and things like this.

P81, social worker, nongovernment community

However, utilization was dependent on the apps’ cultural responsivity to First Nations cultures. This was expressed by 1 participant:

e-health in general, some of them are not [developed for First Nations clients] so we wouldn’t be using it.

P89, psychologist, Aboriginal community−controlled health service

Challenges to electronic mental health utilization in communities, particularly those living in remote areas, were limited availability and accessibility to resources such as IT and the internet as well as low IT literacy levels:

It depends on the situation or the context…a lot of stuff can be difficult in remote communities because of access to internet or things like that.

P98, social worker, Aboriginal nongovernmental organization)

#### Usability and Relative Advantages

Overall, most participants perceived electronic mental health resources to be more interactive, engaging, and useful for bridging literacy gaps as well as enabling clients to exert ownership over their well-being journey:

It was far better than having a therapist really because they could do it at their speed, and it was often someone with anxiety and a bit of OCD and things like that. They have to get things just right and having a computer to deal with is really very, very good for them.

P41, occupation, and organization not reported

One participant also commented that electronic mental health is flexible, easily accessible, and a useful resource:

I do like that you can call a phone number for mental health and you can get advice whether you’re a patient or whether you’re a professional. I like the way you can go online and you can do little tests and go, “Okay. I’m not suicidal” or “Okay. My child’s not depressed.”

P108, general nurse, government community

Specific to the Stay Strong app, some participants expressed that the app has relative advantages over a traditional paper format in terms of data recording and management. They also voiced that it helped streamline their approach and communication between staff:

You can fill out all the information you need on an app and then email your information straight across so it makes life a little bit easier for us.

P124, social worker, nongovernment community

However, the technological requirements of electronic mental health approaches were perceived to impede implementation by most participants. Specifically, participants reported difficulties accessing the appropriate technology, that is, iPads or the internet as well as having trouble with various aspects of the technology such as charging, passwords, and connectivity requirements:

We have challenges with our apps and our iPad because the communities I go to don’t have WIFI hotspots so I can’t access internet.

P108, general nurse, government community

Moreover, for the participants who worked across multiple sites, having to remember to take the technology with them was a challenge:

We didn’t even think to grab the i-Pad when we were heading out, or even ask for it.

P85, alcohol and other drug worker, Aboriginal community−controlled health service

#### Trialability

Having a good understanding of how electronic mental health resources are effectively used in a real-life context was identified to be important in the training and implementation process.

So it’s all good and well to role play or simulate using it, but until you have the client there – especially with a young person whose vocabulary isn’t the same as an adult, you know, that’s where more of the training would happen.

P205, youth and family worker, nongovernment community

One participant reported having undergone a trial of electronic mental health resources with their clients to assess suitability:

We trial them on a small group of people to see if it’s suitable for our population and see where we go from there.

P124, social worker, nongovernment community

This suggests that, at least for this organization, electronic mental health approaches can be implemented in a step-by-step process.

### Recipient

The definition of recipient is as follows: “Actors involved in, affected by, and influencing implementation. Characteristics of the individuals, eg, motivation, values, beliefs, skills and knowledge, and factors relating to the team’s culture influence individuals and teams in supporting or resisting an innovation.”

#### Motivation

In general, most participants expressed enthusiasm toward electronic mental health approaches; however, some participants commented that having the motivation to incorporate it into practice was a challenge. The lack of motivation was partly due to high workload:

People are already feeling overwhelmed by their work and then all of a sudden, “Oh, I’ve got to do it this way”, because you have to put more thought and effort into it and that turns people off.

P133, other nurse, government community

Nevertheless, motivation was acknowledged to be intrinsic, which could overcome the challenges of circumstance. As one participant said:

Your barrier is yourself... when you’ve got a hundred other things that you’re focusing on with a young person, adding another one in often seems like a barrier but it isn’t.

P193, youth and family worker, nongovernment community

#### Values and Beliefs

Electronic mental health approaches were perceived by the majority of the participants to fit with the times and future directions and to be particularly applicable for young people:

More relevant in peoples' modern lives. It's using technology these days, and if we can keep using paper based and forms that are wordy, that sort of thing, we are not going to be effective so yes, I think it's good. It's simple language and it's digital which is where we need to be.

P129, peer support worker, government community

It was also considered to be a good and valuable option for therapeutic practice. However, 1 participant was concerned that the utilization of electronic mental health resources without professional supervision would negatively impact the face-to-face treatment process:

Some of these e-Health resources adopt psychological procedures [that may not be] appropriate for the client, [so] the client’s attribution is, “Oh, it doesn’t work”. So when they eventually are referred to see a psychologist... it spoils their chances of benefitting from psychological therapy because they come in with a negative attitude already.

P84, psychologist, organization not reported

Successful implementation of electronic mental health was also perceived to be dependent on the organization’s appraisal of the approach. One participant expressed that integration of electronic mental health into the general orientation is dependent on whether the organization saw the value of the approach.

#### Goals

Specific to the Stay Strong app, some participants reported having trouble remembering using it in their day-to-day practice. An explicit goal setting of where, when, and with whom the electronic mental health resource will be used was suggested by 1 participant as a potentially useful technique to combat this issue:

I had these big ideas. I was like, “Oh, I’m going to use it. It’s going to be great. I’m going to get all these really good results” [but]when I do home visits, I…forget to take it with me … – you’ve almost got to allocate a scheduled time of who you want to do it with and say, “Okay. So I’m going to do it on this time.”

P95, mental health support worker, nongovernment community

#### Skills and Knowledge

The lack of knowledge around electronic mental health approaches poses a barrier to utilization. This includes uncertainty around the effectiveness, accessibility, and applicability of the tools and resources. Having the ability to efficiently sort out information and select the right tools and resources for target clients was perceived by 1 participant to be an important skill in electronic mental health adoption:

There is some great resources out there but I need to know where to access them, how to access them, and what to do with them once I have them... Because there is so much out there as well. It's a bit overwhelming at the same time. So to try and pick the right resource for the right setting, for the right people and finding that online, can be a daunting experience as a practitioner in trying to work out where to start with that.

P81, social worker, nongovernment community

Low levels of IT literacy pose a challenge to electronic mental health adoption. Unfamiliarity with different ways of using technologies impedes the utilization of the approach by both service providers and community members. Poor IT literacy within communities was attributed to limited access to technology:

… so many of the patients we work with don’t use technology in that way. A lot of people use mobile phones … not necessarily Smart Phones. Some might enjoy the i-Pad and being able to play games on it, but … accessing computers and the internet within the mob that we work with is not so much.

P91, administration support, Aboriginal community−controlled health service

Finally, the lack of confidence and skills to conduct therapeutic interventions was perceived to impede service providers’ use of electronic mental health resources. For example, specific to the Stay Strong app, 1 participant reported that alcohol and other drug workers were reluctant to use the app as they were not:

comfortable about intervening in a more therapeutic way.

P89, psychologist, Aboriginal community–controlled health service

#### Time, Resources, and Support

Finding the time to explore and learn more about electronic mental health resources was a challenge for many participants. High workload was reported to also delay senior staff, who was perceived to be an important driver, to action implementation:

You need to have will coming from the top…It starts there. Why is there no will? Partly because that person may have so many things… so many battles from the front that something like this falls behind.

P84, psychologist, organization not reported

The lack of IT resources prevented both service providers and community members from using an electronic mental health approach. Many participants reported limited resources such as iPads within their workplace:

I tried to get our iPad – a single iPad that we have here – and it was lost or someone had it and it was just difficult

P122, general nurse, government community

And a lack of IT infrastructure within communities:

Places that young people and older people can go and get online, that is actually going to make a bit of a difference.

P60, Aboriginal community worker, Aboriginal community−controlled health service

Lack of supervision due to skilled staff shortage served as a barrier for incorporating electronic mental health into the general practice:

It was a time when we had very few therapists and so the AOD workers were mostly left to their own devices to interact with clients as they saw fit and we weren’t able to provide closer supervision and so while we did roll out the app, it was seeds not going into fertile ground at that time.

P89, psychologist, Aboriginal community−controlled health service

Insufficient support and training within the organization were also perceived to hinder utility and negatively impact fidelity of use:

The staff, they are not getting enough support, or they might need more training or support, or workshops internally to use it adequately.

P94, manager/coordinator/chief executive officer nongovernment community

#### Collaboration and Teamwork

Some participants expressed the view that staff encouraging, teaching, and holding each other accountable with regard to the use of electronic mental health may facilitate adoption. For instance, 1 participant commented that collaboration and encouragement could prompt utilization.

#### Power and Authority

Staff in an authoritative role were considered important in driving successful implementation. This was highlighted by 1 participant who attributed nonutilization of electronic mental health to the absence of clear directions from those in power:

If the co-ordinator sat down and said, “Okay. We’re going to apply this [the app]…”, then obviously we would follow it through–that would be something that we could focus on - but that was never forwarded to us in any way.

P20, social support worker and well-being, nongovernment community

### Local Context

#### Formal and Informal Leadership Support

Having leaders within the organization showing interest and providing direct support was perceived to facilitate uptake. It created incentives and provided opportunities for service providers to reflect and evaluate the utility of the electronic mental health approach.

The manager was supportive of the app… she would ask how we were going with the application and we would sit down and show her that it is really working and purchasing some results.

P224, manager/coordinator/chief executive officer, Aboriginal community−controlled health service

However, 1 participant reported that too much advocacy could create pressure instead of being supportive:

Definitely supported to use it... There was a lot of – I don’t know. I would say a lot of pressure. Like, “Have you used your Stay Strong yet?”

P121, occupation, and organization not reported

#### Evaluation and Feedback Processes

As illustrated above, evaluation of the effectiveness of electronic mental health approaches may incentivize practitioners to continue using the approach and support fidelity.

### Organization Context

#### Organizational Priorities

Delays in the implementation process were reported to be partly due to resource constraints, that is, technology, workforce, and structures to support electronic mental health approaches. Prioritization of financial and training investment was also perceived to impact implementation. Decisions on which aspects within the organization are worth investing in may render insufficient resources for some programs.

Whether our program has the financial resources to invest in that, I’m not sure. And also whether we are going to have sort of an iPad available for all programs to use which would make it more accessible for my program. And that is still undecided yet.

P149, trainer/educator, government community

Strategic investment in training may potentially assist organizations in sustaining implementation efforts in the long term. For example, one participant reported:

We thought training the local Aboriginal staff was a better option... they are the more appropriate people to talk to their own community... and they are going to be there longer. So in that way, we tried to overcome some of the turnover and issues.

P135, manager/coordinator/chief executive officer, government community

#### Culture

A work culture that values on-going staff development was identified as important for successful implementation. The commitment to upskilling staff may render organizations more receptive to innovations and more inclined to invest effort into the implementation process.

We’re quite keen on the AOD workers up-skilling in their abilities to at least do brief interventions. So when it comes time to concentrate on that, that will be where the app comes into its own and having had it introduced into the team and then some follow-up recently, it will make implementation quite easy.

P89, psychologist, Aboriginal community-controlled health service

A culture of accountability was expressed by one participant to be potentially useful for enhancing adoption:

We have very little accountability generally in our workplace which I would like more of... accountability from management would be good... And perhaps even the peer accountability between the dietitians.

P157, nutritionist, government community

#### Structure and Systems

Two main aspects of the organizational structure and systems were identified as influential in electronic mental health adoption. One aspect relates to the adequacy of the existing (or the lack thereof) structure and systems to support electronic mental health utilization, and the other involves the effectiveness of the integration process in the existing system.

The three key areas identified to be lacking in the organizational structure and systems were resources, policies, and procedures to support the use of the electronic mental health approach. Insufficient IT resources were highlighted as an impediment to utilization. Many participants expressed difficulties in obtaining iPads in a timely manner and gaining access to the internet, particularly in remote settings. In addition, shortage of skilled staff and high staff turnover meant difficulties retaining knowledge and expertise within the workplace, and thus limited support and supervision for the general staff. One participant attributed these issues to inadequate funding, particularly for First Nations organizations:

What’s happening is that we are living in an economic environment where we get funding for six months or twelve months and we’re not attracting staff to stay here or people who can help with the training and the knowledge base of the team…there are a lot of indigenous organisations who are constrained by those very factors.

P89, psychologist, Aboriginal community−controlled health service

Policy was another structural issue identified to influence the utilization of the electronic mental health approach. Inadequate policies regarding the use and access of the technology and the client’s confidentiality were reported to delay the implementation process:

So the policy and procedure needed to be created to get around our usage and downloading apps; and how the staff could access it; and who they need to go to if they found an app that they would like to use.

P149, trainer/educator, nongovernment community

Organizational processes to streamline the integration of electronic mental health into the existing system were identified to be essential for successful implementation. Inadequate integration may increase workload or render the innovation lost within the system, and thus impede staff from incorporating the approach into their practice. In addition, insufficient processes at the higher management level was reported to delay the implementation process and constrained utilization:

We’ve just been held up by, you know … at the Director level … somewhere in the corporate communications area…it’s getting approval for these things.

P134, public health nutritionist, government community

Many participants reported difficulties obtaining iPads in a timely manner due to reasons such as unavailability and empty battery as well as remembering to use it as part of their practice. To address some of these difficulties, one participant suggested developing on the ground processes to systemize the utilization of IT resources:

[the] process could be streamlined…say, “Here – here’s an iPad”– or whatever the process is.

P122, general nurse, government community

Finally, having a procedure to train new staff and disseminate new resources among existing staff was perceived to be important for effective use of electronic mental health in the long term.

I think there should be a process for it so if something new comes out, you tend to go around and show people how they use it and practise it. So you’ll teach someone your bad habits... I think better processes and having some evaluation of it. “We use this because it’s been shown to …” you know, “do this or do that” – not just “Isn’t this pretty and here’s another thing to use”.

P138, general nurse, government community

#### History of Innovation and Change

The history of introducing technology to the workplace may ease the integration of the electronic mental health approach into the system. For example, 1 participant reported:

IT policies are pretty up to date with regards to using iPads and stuff like that, which all happened when we did start getting the iPads in to do – ‘cause we used them for Survey Monkey to do feedback.

P188, case manager, nongovernment community

### External Context

#### Policy Drivers and Priorities

The importance of the external health system in the successful implementation of electronic mental health approaches was foreshadowed throughout. As described in the previous elements, participants expressed the importance of electronic mental health approaches to be validated for the First Nations, IT infrastructure within community and organization, and human resources in electronic mental health uptake. This suggests a perceived need for policies to cover a range of areas, that is, the accreditation process of resources and tools, investment in technology in remote areas, and funding to First Nations organizations.

#### Interorganizational Networks and Relationships

Communication across services working with the same client may help to ensure nonoverlapping of interventions and resources. The issue of repetition posing a negative impact on the clients’ experience of electronic mental health approaches was expressed by 1 participant:

Everybody is coming on board with these apps, and often we all service the same clients…How often do these clients get asked to do this because the clients that we work with often… it’s passive resistance…I wonder if it’s [us] asking them to do the same thing.

P106, occupation and organization not reported

## Discussion

### The Integrated Promoting Action on Research Implementation in Health Services as a Determinant Framework

To further understand the conditions for successful implementation of electronic mental health approaches, this study aimed to provide insights into factors that impede or facilitate electronic mental health uptake within primary health care services for Australia’s First Nations in the Northern Territory. The *i*-PARIHS was used as a determinant framework to deepen our understanding and interpretation of service providers’ experiences and perspectives on electronic mental health adoption.

The framework had been previously used in a similar manner—as a retrospective analysis by Laycock et al [40], who found it to enhance their understanding of factors that influenced the interactive dissemination process. Similarly, we found that the framework helped us to understand service providers’ experiences systematically. It also deepened our understanding of how the *innovation*, *recipient*, and *context*, relating to electronic mental health adoption, operationalized in a real-life context. However, we also found that some of the *i*-PARIHS elements could not be analyzed as separate constructs due to their interdependencies. This is due to both the complex nature of implementation, that is, the inter-relations and flow-on effects among factors in determining implementation outcomes as well as the difficulty in teasing out conceptual differences among some of the *i*-PARIHS subelements.

For example, the lack of human resources and IT infrastructure at the organizational level (*context*) impacted the availability of supervision and technological devices for individual practitioners (*recipients*). Therefore, when discussing the negative impact of the lack of resources and support on electronic mental health uptake, it is difficult to categorize if its related to *context* or *recipients* when there is an interdependency, that is, the lack in the organizational structure has a flow-on effect on the individual practitioner.

Another example is the issue of knowledge related to electronic mental health approaches. Participants expressed a lack of knowledge and confusion regarding the context and the population for which electronic mental health approaches were developed and validated. Thus, they were uncertain about the suitability of the resources for their clients. This important issue is related to *recipients* as it highlights that, practitioners themselves lack the knowledge; however, it is also related to *innovation* as it demonstrates the importance of information from research, clinical, patient, and local experience in electronic mental health uptake. Moreover, there were no definitions for the subelements within each of the core elements, and thus we had to rely on our understanding.

With regard to the conceptualization of *facilitation* (an active ingredient that assesses and aligns *innovation*, *recipients,* and *context*), we found it to provide greater depth to our implementation strategy. We viewed ourselves as an external facilitator aiming to empower internal facilitators and service providers to incorporate electronic mental health as part of their practice. We intend to use our findings relating to the *innovation* (electronic mental health approach), *recipients* (service providers as an individual and as a team), and *context* (local, organizational, and external contexts) to inform future facilitation strategies and catalyze electronic mental health uptake.

The key findings, as discussed below, were therefore used to develop a structured approach or framework for implementing electronic mental health approaches within First Nations primary health care services.

### Principal Findings

Similar to previous studies, service providers showed optimism and enthusiasm for electronic mental health approaches and viewed them as valuable and potentially useful resources, particularly for young people [32,33]. In addition, the approach was perceived to be suitable and useful for First Nations communities, resonating with previous findings from other health organizations and community members [29,33]. With the omnipresence of information and communication technology in today’s society, digital media such as smartphones and computers are inevitably becoming part of and shaping First Nations’ lives [26,27]. Although still in its infancy, the literature suggests promising potential for electronic mental health to enhance First Nations well-being and culture [41,42].

However, limited availability and access to IT resources in some areas impeded electronic mental health utilization and contributed to community members’ low levels of IT literacy. The lack of resources, particularly in remote areas, is recognized by the Australian Government. Efforts are being made under the Mobile Black Spot Program and the National Broadband Network as part of the government *Closing the Gap 2016* initiative to ensure the availability of IT across Australia [43].

In line with findings from systematic reviews on factors essential to the successful implementation of electronic mental health approaches [44,45], the present findings demonstrate the importance of service providers’ knowledge, skills, and perception in electronic mental health adoption. Particularly, service providers’ low levels of IT literacy pose a challenge to electronic mental health utilization. The importance of IT proficiency in electronic mental health adoption was highlighted in previous studies in primary health care for First Nations. This suggests the potential usefulness of basic IT skills training for service providers [32,33]. In addition, service providers had limited knowledge of electronic mental health approaches, particularly of the context in which the resources were developed, the population they were developed for, and whether they have been validated. This resonates with the general lack of electronic mental health awareness and knowledge exhibited by the wider population [24,46,47].

This lack of knowledge impeded utilization, as confidence that the resources are appropriate for First Nations and the local settings was essential to uptake. The need for electronic mental health resources to be culturally responsive to First Nations has been previously highlighted [29,33]. As part of the electronic mental health training program provided by Menzies, culturally responsive tools and resources such as the Stay Strong app are promoted. The training program also promotes directories, such as Head to Health and Beacon, which provide links to other relevant resources. Nonetheless, future adaptation of the training program will develop service providers’ skills in obtaining and evaluating relevant information on electronic mental health approaches to facilitate independent selection of suitable tools for their clients.

The adoption of electronic mental health was also dependent on the perceived fit of the approach to the organizational ethos as well as on the practitioners’ role and responsibilities, which is in accordance with previous findings [32,33,44,45]. More specifically, service providers who held managerial roles, and those who did not work with clients in a therapeutic way did not use the approach. However, it was recognized that leaders within the organization, who may not work with clients directly, can have a positive impact by championing the approach and influencing others to use it as part of their practice. This resonates with the literature that identifies internal facilitators as important figures in successful implementation [48,49].

Similar to the systematic reviews’ findings, we found that organizational factors played a major role in electronic mental health uptake [44,45]. In particular, the lack of IT resources and infrastructure posed a significant barrier to utilization. Insufficient workforce due to staff shortage and high staff turnover was an impediment as it meant high workload and difficulties retaining knowledge and expertise within the workplace. Uptake was hindered by inadequate policies to guide the appropriate use of technology and confidentiality, and insufficient processes to streamline the integration of electronic mental health approaches into existing systems. Direction and support from leaders in the organization were also important for providing incentives and may enhance fidelity in electronic mental health use. These barriers were previously noted by Bennet et al [32].

A scoping review found that organizational drivers (decision support, administration, and system intervention) and leadership drivers received significantly less attention than competency drivers (staff and user selection, training, and supervision) in the electronic mental health implementation literature [50]. The importance of organizational structure, systems, and leadership support in electronic mental health adoption highlighted in the present findings as well as in the previous findings [32,33] suggest that much more attention to these elements is needed.

It is important to note that Puszka et al [33] reported on chief executive officers’, mangers’, and directors’ perspectives on potential barriers and enablers of electronic mental health implementation, whereas this study provided the experiences of service providers who have undergone electronic mental health training and had some time to apply what they have learned into their practice. Nonetheless, similar issues around organizational readiness have been raised by both cohorts, thus emphasizing the need for organizational readiness to be addressed in implementation strategies.

Organizational readiness is conceptualized to comprise *both individual difference and structural factors, reflecting the extent to which the organization and its members are inclined to accept, embrace and adopt a particular plan to purposefully alter the status quo*. Individual difference refers to the *characteristics of those being asked to change* (eg, skills, knowledge, attitude, perception), and structural factors refer to *the circumstances under which the change is occurring* (eg, infrastructure, policies, resources availability) [51].

The importance of organizational readiness in successful implementation is well recognized in the literature. Multifaceted approaches to implementation are considered an optimal strategy for influencing change [52]. In the literature, implementation frameworks such as the four-stage implementation model developed by the National Implementation Research Network [53] and the Quality Implementation Framework [54], propose multiple stages to implement with the initial stages, focusing on need analysis and development of strategies addressing organizational readiness.

Reflecting on our electronic mental health implementation strategy, we recognize the limitations of one-off trainings and the need for a more comprehensive facilitation strategy that addresses organizational readiness. Taken together, the present findings and the literature, we propose a multistaged implementation strategy aimed at enhancing electronic mental health uptake by addressing the different aspects of organizational readiness relating to electronic mental health implementation at both the individual and structural levels.

Given the importance of preparatory stages as outlined in other existing implementation frameworks, for example, the National Implementation Research Network and The Quality Implementation Framework [53,54], we propose a three-phase implementation strategy for electronic mental health implementation in health care, involving: (1) pretraining consultations to address organizational readiness, (2) training workshops to increase awareness, knowledge, and skills relevant to electronic mental health utilization, and to train internal facilitators to supervise, support, and conduct trainings within the organization, and (3) follow-up support to boost the trainees’ motivation and the organizations’ efforts to incorporate electronic mental health into practice and the existing system, and to provide support for internal facilitators in supervising and training other staff.

#### Pretraining Consultations

Our findings demonstrate the importance of organizational readiness in successful implementation. Organizational factors highlighted as essential to uptake are IT resources and infrastructure; policies around the use of technology and confidentiality; human resources; processes to streamline integration of electronic mental health into the existing system; and leadership/internal facilitators to advocate, action implementation, train, and provide support and supervision for on-going electronic mental health utilization within the organization. Thus, before training, it is pertinent for organizations to assess their level of readiness across the highlighted areas to identify aspects that require improvements. Key considerations are listed in Textbox 1. To ensure that organizations have thoroughly reflected on their readiness and improve the identified areas, we propose at least two consultation sessions. The objective of the first session is to aid organizations to assess their level of electronic mental health readiness and develop an action plan to address areas in which adaptation is required. The second session’s aim is to provide an opportunity for further identification and refinement of strategies to address the areas of improvement.

This stage should also instigate reflections on who within the organization should attend training and who would be appropriate to carry the role of an internal facilitator. Moreover, information regarding organizations’ local settings and target populations, as well as the service providers’ levels of IT skills and literacy, should be noted. This information would be useful in informing the training sessions so that the resources and information are contextualized and more relevant and useful for trainees.

**The three-phase facilitation process for electronic mental health implementation.**

Phase 1: Pretraining consultations

Consultations assess the following aspects of organizational readiness:

- Information technology (IT) resources and infrastructure, that is, computers, iPads, and Wi-Fi connectivity
- Policies and procedures to guide collection, confidentiality, and security of data
- Current conducive work culture and climate (eg, accountability, on-going learning, staff turnover, and priorities)
- Staff skills and literacy in IT and electronic mental health approaches
- Suitability of client population and cultural context
- Internal facilitators to champion, train, and support on-going e utilization

Phase 2: Training

Workshops occur when suitable organizational readiness is achieved through mutual agreement and incorporate the following elements:

- IT skills training if needed (determined through consultation)
- Presentation of electronic mental health resources relevant to organizations’ local and cultural needs
- Strategies to find and evaluate electronic mental health resources
- Information regarding electronic portals hosting resources that are filtered by experts
- Planning when, where, and with whom, electronic mental health resources will be used
- Training for internal facilitators to supervise and support on-going electronic mental health use

Phase 3: Follow-up support

- Face-to-face, email, and telephone follow-up to support on-going use
- Face-to-face, email, and telephone follow-up for internal facilitators to support their training, support, and supervision
- Review progress of organizational readiness and support implementation
- Guidelines for evaluating the implementation strategy
- Assessment of organizational readiness before, during, and postintervention. The assessment should cover individual characteristics as well as structural factors, as discussed in the literature [51]. The assessment should function as both a diagnostic and evaluative tool to track the trajectory of organizational readiness over the course of the intervention
- Comparison of the frequency of electronic mental health use before and after the intervention
- Frequency and quality of trainings conducted within the organization by internal trainers
- Service providers’ skills, knowledge, and perception of electronic mental health approaches before, during, and postintervention
- Records of discussions during pretraining consultations and follow-up support to illuminate the progression and type of support needed throughout the intervention

#### Training

The training should be contextualized and involve information and activities relevant to the trainees. That is, it should include resources that are applicable to the trainees’ local settings and target populations, that is, resources that are culturally relevant to local indigenous populations as well as incorporating IT training to ensure adequate levels of IT literacy for electronic mental health utilization. The training should also provide trainees with the skills to find, assess, and evaluate information to select the right electronic mental health resources for their specific clients. In addition, a time should be set aside for implementation planning, so trainees can plan where, when, and with whom they would use an electronic mental health approach.

A separate training for the selected individuals who would take on the internal facilitator role within the organization should be offered. Specifically, it should provide internal facilitators with the knowledge and skills to support, supervise, and conduct electronic mental health training with the organization.

#### Follow-Up Support

The literature highlights the importance of follow-up supervision post one-off workshops in influencing therapist behavior and patient outcomes [9,32]. Specific to electronic mental health implementation, Bennett-Levy et al [55] reported the positive impact of post-training consultations regarding the enhancement of skills and exploration of electronic mental health resources. Therefore, follow-up support should be provided to trainees and organizations following training workshops. It should include occasional face-to-face meetings and regular emails and phone calls to motivate on-going use and to address any issues regarding electronic mental health utilization, such as skills and confidence, technical setups, and troubleshooting. In addition, separate support should be provided to the internal facilitators to ensure the fidelity of electronic mental health training and implementation within the organization. Finally, organizational readiness should be reevaluated to identify any further gaps and to ensure the effectiveness of the ongoing progress in the integration of the electronic mental health approach into the wider system.

### Limitations

Our position as an eMHPrac partner as well as developers of the AIMhi Stay Strong app, which is included in the training workshop, may have introduced a positive bias and influenced data collection and the analysis process. However, due to our position, we have established rapport with the participants before the interviews, which may have rendered participants more comfortable in sharing their experiences. In addition, through the delivery of our training program, we have developed an understanding of local service delivery contexts. Nonetheless, we were cognizant of the way in which our subjectivity may have influenced and shaped the different stages in the research process.

The analysis was carried out in two stages upon the discovery of the revised *i-*PARIHS framework. It is possible that starting the analysis with the *i*-PARIHS framework may have led to some nuances in the results. However, the data were initially analyzed inductively, that is, codes and themes were identified without the guidance of any particular framework. Therefore, the data extracts, the initial codes, and themes identified as relevant to electronic mental health implementation were not influenced by either the *i*-PARIHS or PARIHS framework. As this initial stage is strongly informed by data, it is unlikely that the sequence of applying the framework to the data would have majorly impacted the results.

The present findings are consistent with those in the literature regarding factors essential for successful implementation of electronic mental health approaches in health care settings. However, it is important to acknowledge the context of our participants, who are service providers working with Australia’s First Nations in the Northern Territory. Certain factors, such as limited resources, IT literacy, and high staff turnover, may be more salient to this context relative to other services in urban areas working with nonindigenous populations.

### Conclusions

Through the lens of the *i*-PARIHS framework, this study provides a practical understanding of how different factors impede or facilitate electronic mental health implementation within primary health care servicing Indigenous Australians. This insight laid a foundation for a comprehensive facilitation strategy, involving three stages: pretraining consultations, training workshops, and post-training follow-up support, aiming to enhance the success of electronic mental health implementation.

## Abbreviations

AIMhi: Aboriginal and Islander Mental Health Initiative
eMHPrac: electronic mental health in practice
ePortal: electronic portal
i-PARIHS: integrated Promoting Action on Research Implementation in Health Services
IT: information technology
Menzies: Menzies School of Health Research
PARIHS: Promoting Action on Research Implementation in Health Services
QUT: Queensland University of Technology
UCRH: University Centre for Rural Health
